# Data driven filtering of bowel sounds using multivariate empirical mode decomposition

**DOI:** 10.1186/s12938-019-0646-1

**Published:** 2019-03-20

**Authors:** Konstanze Kölle, Muhammad Faisal Aftab, Leif Erik Andersson, Anders Lyngvi Fougner, Øyvind Stavdahl

**Affiliations:** 1 Department of Engineering Cybernetics, Norwegian University of Science and Technology (NTNU), Trondheim, Norway; 2 Department of Endocrinology, St. Olavs University Hospital, Trondheim, Norway

**Keywords:** Bowel sounds, Multivariate empirical mode decomposition, Fractal dimension

## Abstract

**Background:**

The analysis of abdominal sounds can help to diagnose gastro-intestinal diseases. Sounds originating from the stomach and the intestine, the so-called bowel sounds, occur in various forms. They are described as loose successions or clusters of rather sudden bursts. Realistic recordings of abdominal sounds are contaminated with noise and artifacts from which the bowel sounds must be differentiated.

**Methods:**

The proposed intrinsic mode function-fractal dimension (IMF-FD) filtering utilizes the property of the multivariate empirical mode decomposition (MEMD) to behave as a series of band pass filters. The MEMD decomposes the abdominal signal into its different frequency components. The resulting intrinsic mode functions (IMFs) are modulated in amplitude and frequency where transient sonic events occur. Based on the complexity of the IMFs, measured by their fractal dimension (FD) in sliding windows, the information-carrying IMFs are selected. The filtered signal is formed as the superposition of all selected IMFs. The IMF-FD filter not only enhances the non-linear components of the original signal but also segments them from the rest. Another important aspect of this work is that typical artifacts that occur in the same frequency range as bowel sounds can be subsequently eliminated by heuristic rules.

**Conclusions:**

The method is tested on a realistic, contaminated data set with promising performance: close to 100% of the manually labeled bowel sounds are identified.

## Introduction

Classical diagnosis of gastrointestinal disorders is uncomfortable for the patient, time-consuming for the medical staff, and expensive for the health care system. The irritable bowel syndrome, for example, affects up to 16% of the population in the United States and results in costs of $1 billion [1]. Diagnosing gastrointestinal disorders based on manual auscultation is not only time-consuming but also prone to error [2]. This not only increases the health care expenditure but also prolongs the discomfort for the patients. It has also been shown that a dysfunctional digestion correlates with psychological ill-being [3].

Non-invasive monitoring of abdominal sounds by a microphone attached to the abdomen is cheap, and more convenient for both the patients and the medical practitioners alike. The recordings can be analyzed by computer programs. Computerized analysis of abdominal recordings could, for example, help differentiating between the irritable bowel syndrome and Crohn’s disease [4]. The assessment of gastrointestinal motility after major abdominal surgery is another area of application [5]. Recently, abdominal sound monitoring has also been suggested for meal detection in the context of an artificial pancreas [6].

In order to utilize abdominal sound monitoring for such analyses, bowel sounds must be separated from background noise. This paper proposes a data driven filtering method that enhances bowel sounds by removing noise and typical artifacts.

### Paper organization

The paper is organized as follows: In the Background section, bowel sounds are characterized and the processing of abdominal sound recordings that are contaminated by artifacts are outlined. Furthermore, empirical mode decomposition and its advantages are introduced. The next section describes the setup of the data recording. Afterward, the proposed method is explained and illustrated by one exemplary recording segment. Results are reported by means of chosen details to emphasize particular properties of the method and in summary for all tested segments. Following this, the work is discussed and concluded.

## Background

### Characterization of bowel sounds

**Fig. 1:**
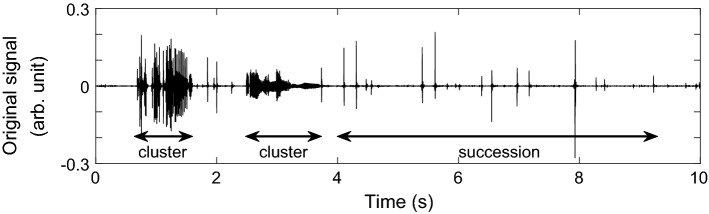
Bowel sounds in clusters and as sparse succession. The raw signal has been processed by a high pass Butterworth filter with a cut-off frequency of 50 Hz to enhance the visibility of bowel sounds in this figure

Bowel sounds are non-stationary, transient events. Two main types of bowel sounds can be differentiated: (a) clicks of short duration occurring alone or in sequence, (b) clusters of (non-differentiable) bursts of longer duration, e.g. [7]. Figure 1 depicts both types. The typical frequency range of bowel sounds lies between 50 and 1500 Hz [8–10]. Single studies report maximum frequencies of up to 3000 Hz [11] or 5000 Hz [12]. However, the power of the signal above 1500 Hz is rather small [11]. A more recent study confirmed that only 0.5% of the signal’s power spectrum density occurs at frequencies above 1000 Hz [13]. The same study revealed that the largest part of the power spectrum density of abdominal sounds is located between 100 Hz and 500 Hz [13].

### Processing contaminated abdominal sound

Artifacts contaminate sound recordings, and their elimination is challenging since the frequency range of typical artifacts, such as from swishing clothes, is the same as that of bowel sounds [13]. For artifact elimination, heuristic rules concerning the duration and energy content of identified sounds have been applied [13]. Statistical separation of bowel sounds and artifacts by means of neural networks has also been suggested [14].

Besides thresholding of the raw audio signal [14] or its envelope [11], mainly wavelet-based methods have been proposed to amplify and extract bowel sounds from background noise. The idea of thresholding the wavelet coefficients proved useful [15] and was further refined [16]. In a different approach, the fractal dimensions of the raw signal were investigated [17]. These two methods have been combined later to using the fractal dimensions of the wavelet coefficients for bowel sound amplification [18].

### Empirical mode decomposition

The complexity and the non-stationary nature of bowel sound signals require special attention and care. An important limitation of wavelet based methods is the predefinition of the basis function used for the decomposition. An adaptive data driven analysis technique, called empirical mode decomposition (EMD) [19], and its variants are finding their ways in a number of technological areas owing to its peculiar properties. In bio-medical data processing, it has been applied to electroencephalography (EEG) signals [20] and foetal heart rates [21]. The EMD process decomposes the signal into modes called intrinsic mode functions (IMFs). Unlike the wavelet decomposition, the EMD basis functions (the IMFs) are calculated from the data itself. An important property of the EMD is the dyadic filter bank property by virtue of which it behaves as a series of band pass filters [20].

This paper utilizes the filter bank property of the EMD to detect bowel sounds. After decomposing the signal into intrinsic mode functions, their fractal dimension (FD) is used as criterion for selecting the information-carrying IMFs. By this novel combination of EMD and FD analysis, both the background noise is reduced and single bowel sounds are segmented.

## Experimental setup

### Recording

A condenser microphone of type Sennheiser MKE2 P-C was fixed in the center of the chest-piece of a classical stethoscope. Medical tape was used to attach this modified chest-piece in the upper right quadrant of the abdomen. The audio signals were recorded with a sampling frequency of 32000 Hz and a resolution of 24-bit using a the digital audio recorder 722 (Sound Devices LLC, Reedsburg, Wisconsin, US).

**Fig. 2:**
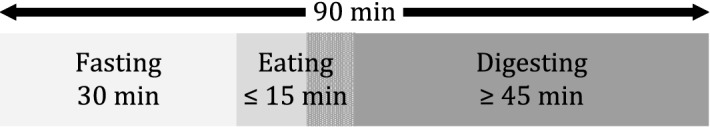
Protocol of recording sessions

The Regional Ethical Committee Central approved this pilot study, REK midt 2018/2028. Ten volunteers were enrolled that reported themselves as healthy with respect to gastrointestinal functions. One recording was generated for each of the ten subjects. During the 90 min-recording sessions, the subjects remained silently seated in a reclined position with elevated legs and refrained from movements as much as possible. The lunch meals were positioned at an easily reachable distance from the subject. As Fig. 2 illustrates, the subjects were fasting in the first 30 min of the experiment. After this fasting interval, the subjects ate for a maximum of 15 min.

### Pre-processing

The raw signals were down-sampled to a frequency of 4000 Hz using a Chebyshev Type I IIR filter of order 8 by means of Matlab’s built-in function decimate. This accelerated the processing without loss of information since the maximum frequency of bowel sounds is not higher than 1000 Hz as discussed earlier.

### Data exclusion

Two of the ten recordings were excluded: One recording contained almost no variations or audible sounds, respectively. This could be caused by poor contact between the skin and the microphone. The other recording was excluded because of very severe contamination with background noise, originating possibly from electrical interference.

### Segmentation and labeling of test set

A test set with labeled bowel sounds was generated to test the proposed method. From the eight included recordings, 10 segments, each with a duration of 10 s, were extracted. In particular, two successive segments were extracted 15, 25, 35, 45, and 55 min after the beginning of each recording (15:00–15:10, 15:10–15:20, 25:00–25:10, 25:10–25:20, 35:00–35:10, 35:10–35:20, 45:00–45:10, 45:10–45:20, 55:00–55:10, 55:10–55:20). This distribution was chosen to include both the fasting and the digesting state.

Bowel sounds in these eighty 10 s-segments were identified by audio-visual examination using version 2.1.0 of Audacity^®^ [22], labeled and exported as text-files. The labels were imported into Matlab and compared with the bowel sounds that were identified by the intrinsic mode function-fractal dimension (IMF-FD) method described in the following.

## Methods

The proposed filtering method was developed using data from one additional subject not part of the eight subjects in the test set. The method is illustrated in the following on the basis of the segment shown in Fig. 1.

### Multivariate empirical mode decomposition (MEMD)

The empirical mode decomposition (EMD) is an iterative, data driven decomposition method. Central to the EMD are the intrinsic mode functions (IMFs) which have zero mean and a number of extrema and zero crossings that differ at most by one. The IMFs are adaptively sifted out from a univariate signal beginning with the fast frequency components. Cubic splines are fitted as envelopes through local extrema. The local means of these envelopes describe the low frequency components. The local fast components are obtained by subtracting the local means from the signal. This is repeated on the identified local fast components until the remaining fast components comply with the above definition of an IMF. The residue of the original signal and the IMF is iteratively processed in the same way until no further IMF can be extracted from the residual. By adding all *M* IMFs and the residue ${\textit{res}}(t)$, the original signal *x*(*t*) can be obtained [19]:

1 $$\begin{aligned} x(t) = \sum _{m=1}^M IMF^m(t)+{\textit{res}}(t)\,. \end{aligned}$$

**Fig. 3:**
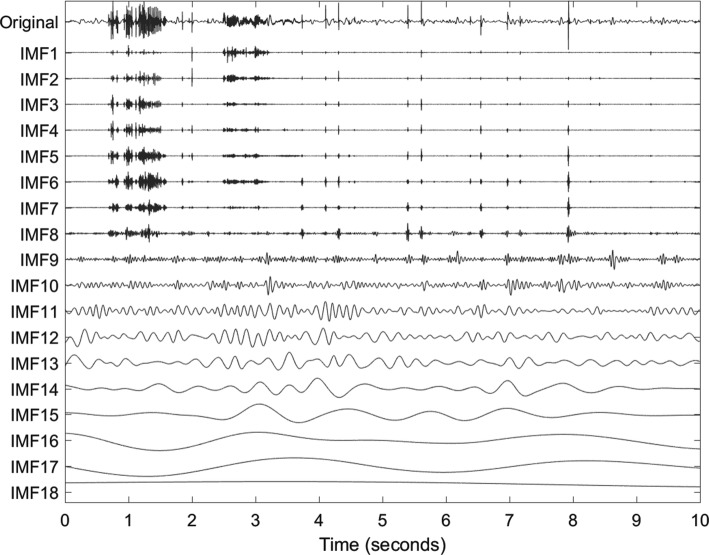
Original signal (same as in Fig. 1) and its decomposition into IMFs by applying MEMD. All *M* IMFs are individually scaled to the range $\text {max}(\left| IMF\right| ) \cdot \left[ -0.1,0.1\right]$

The multivariate EMD (MEMD) is an extension of the EMD to multivariate signals. The noise-assisted version of the MEMD reduces mode mixing within IMFs of a single channel [20]. Therefore, the mono-variate signal is converted to a multivariate signal by adding two channels of white noise before it is decomposed by an MEMD. The IMFs corresponding to the noise channels are then discarded before further processing. Figure 3 shows the IMFs after decomposing the signal in Fig. 1 using MEMD. The first eight IMFs clearly resemble the sonic events in the original signal by larger amplitudes.

**Fig. 4:**
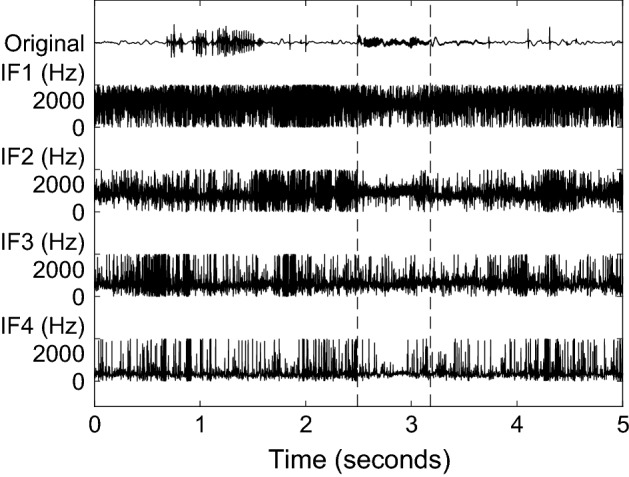
Original signal (same as in Fig. 1) and instantaneous frequencies (IFs) of the first four IMFs in Fig. 3. The first five seconds are shown. Vertical dashed lines indicate the discussed sonic event from 2.5–3.2 s

An IMF represents one mode of oscillation without riding waves. Non-stationarities in the decomposed signal result in amplitude- and frequency-modulated IMFs. The instantaneous frequencies (IF) can therefore change within one IMF. Figure 4 shows the IFs of the first four IMFs in Fig. 3. A spike in these signals represents abruptly changing frequencies in the underlying IMF. The IFs of the first IMFs fluctuate heavily. At times with sonic events, e.g. from 2.5 s to 3.2 s, IF2 fluctuates less. Comparing IF2 with its IMF in Fig. 3, one can see that IMF2 has large amplitudes at sonic events such as between 2.5 s and 3.2 s, but is close to zero otherwise. The heavy fluctuations of IF2 can be accredited to noisy components.

### Dyadic filter bank property of MEMD

**Fig. 5:**
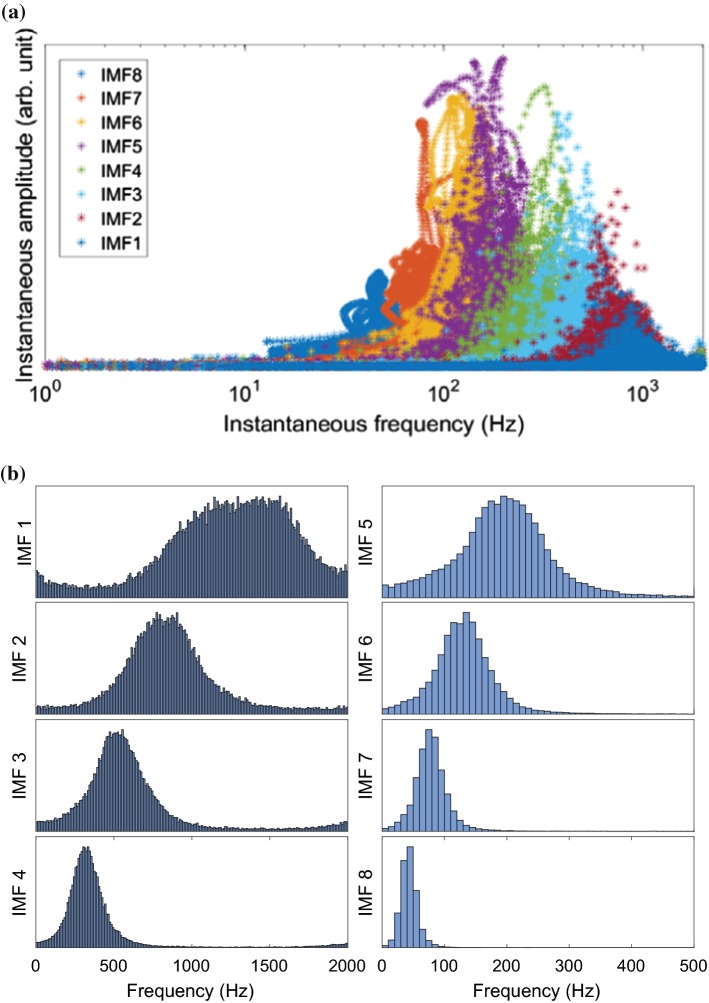
Spectral distribution of the IMFs (distribution of instantaneous frequencies) in Fig. 3. **a** Spectral distribution of the first eight IMFs in Fig. 3 illustrating the dyadic filter bank property of MEMD. **b** Histograms with 200 bins of the spectra of the first eight IMFs in Fig. 3

An important property of MEMD is its filter bank property, where it behaves as a series of band pass filters [20]. This dyadic filter bank property is illustrated in Fig. 5a. Each marker represents the instantaneous amplitude of the instantaneous frequency at one time instance of one IMF. The spectrum of an IMF is not a sharp peak at a single frequency but spreads over a range of frequencies. The spectra of successive IMFs are overlapping.

The spectra of the first eight IMFs in Fig. 5a are plotted as histograms in Fig. 5b. The histograms show the rate of occurrence of each frequency; information of the amplitude is omitted. IMF1 has a wide frequency distribution with no distinct peak. Moreover, the plateau appears between 1000 and 1600 Hz, and thus, at frequencies outside the range of bowel sounds. All together, this IMF seems to contain mostly noise or spurious components. With an increasing IMF index, the spectra are shifted to lower frequencies and show more distinct peaks.

### IMF-FD filter to enhance bowel sounds

#### Selecting information-carrying IMFs based on their fractal dimension

As we saw in Fig. 3, some IMFs mirror the variations of the original signal more than others: changes in IMFs 1–8 correlate with the sonic events in the original signal, whereas the other IMFs have no obvious relation to the sounds.

The complexity of a waveform signal can be described by its fractal dimension. For identification of bowel sounds, fractal dimension analysis has been applied directly to the sound signal [17] and to coefficients of the wavelet transform [18], respectively. Inspired by the latter, we analyze the fractal dimension of a *K*-sample signal in sliding windows of length $W_L = 0.0006 \cdot F_s+1$, with $F_s$ being the sampling frequency. The $K-W_L+1$ windows are shifted by one sample, and the FD values are assigned to the centers of these windows.

For each IMF, the fractal dimension is approximated in the $K-W_L+1$ windows using the definition by Sevcik [23]:

2 $$\begin{aligned} F\!D= 1 + \frac{\ln {L_c}}{\ln {\left[ 2\cdot (W_L-1)\right] }} \ , \end{aligned}$$

where $W_L$ is the length of the window, and $L_c$ is the sum of Euclidean distances between successive signal samples $l_i$:

3 $$\begin{aligned} L_c = \sum _{i=1}^{W_L-1} \left\| l_{i+1}-l_i\right\| \ . \end{aligned}$$

**Fig. 6:**
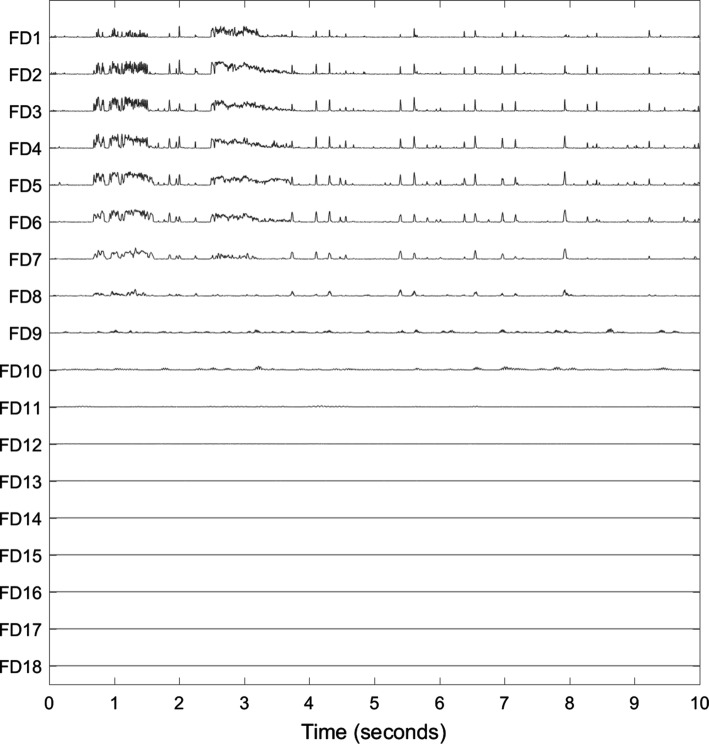
Local fractal dimensions (FDs) of the IMFs in Fig. 3. The same scaling was applied to all FDs

Figure 6 presents the sliding FDs of the IMFs in Fig. 3. If the signal was flat in a window with 25 samples ($W_L = 25$) and a sampling rate of $F_s = 4000$ Hz, it would have the minimum FD:

4 $$\begin{aligned} F\!D_{\text {min}} = 1 + \frac{\ln {\left[ (25-1) \cdot 1/4000\right] }}{\ln {\left[ 2\cdot (25-1)\right] }} = - 0.3216 \ . \end{aligned}$$

The notation $F\!D^m$ indicates the vector containing the local fractal dimensions of the *m*-th IMF; $F\!D^m_k$ is the $F\!D^m$ at sample *k*. Mean $\mu ^m$ and standard deviation $\sigma ^m$ of $F\!D^m$ are defined as:

5 $$\begin{aligned} \mu ^m = \frac{1}{K} \left( \sum _{k=1}^K F\!D^m_k\right) \ , \end{aligned}$$

6 $$\begin{aligned} \sigma ^m = \sqrt{\frac{\sum _{k=1}^K \left( F\!D^m_k-\mu ^m\right) ^2}{K-1}} \ . \end{aligned}$$

**Fig. 7:**
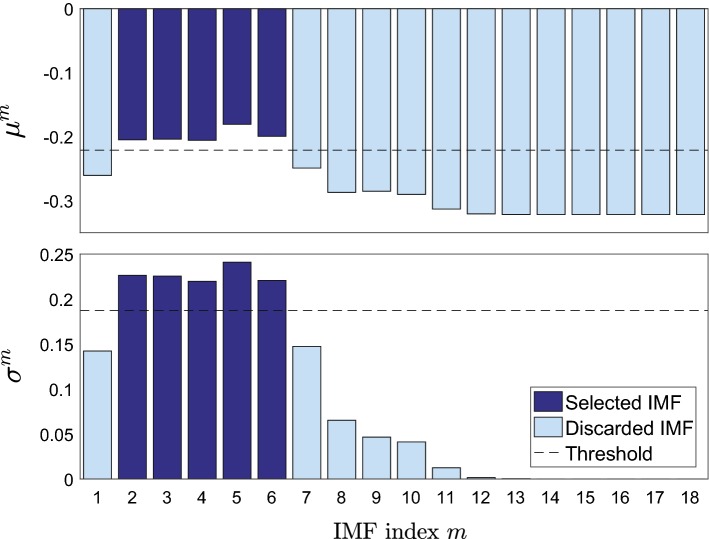
Selected IMFs. Selection of IMFs by thresholding the mean $\mu ^m$ and standard deviation $\sigma ^m$ of the fractal dimensions

They are illustrated in Fig. 7 and used to measure the variation of IMFs. Those IMFs carrying more information about the non-stationary bowel sounds, have higher means and standard deviations.

The *M* IMFs are sorted into the set of selected IMFs ($\mathbb {M}_S$) and the set of discarded IMFs ($\mathbb {M}_D$). These sets are defined as:

7 $$\begin{aligned} m \in {\left\{ \begin{array}{ll} \mathbb {M}_S &{}\text {if}\,\, \mu ^m \ge \mu (\mu ^M) + \sigma (\mu ^M) \\ \\ &{} \quad \wedge \quad \sigma ^m \ge \mu (\sigma ^M) + \sigma (\sigma ^M)\\ \\ \mathbb {M}_D &{}\text {otherwise,} \end{array}\right. } \end{aligned}$$

where $\mu (\mu ^M)$ and $\sigma (\mu ^M)$ are the mean and standard deviation of all *M* $\mu ^m$, and $\mu (\sigma ^M)$ and $\sigma (\sigma ^M)$ of all *M* $\sigma ^m$, respectively. The threshold on $\mu ^m$ selects the IMFs with on average high FD. The threshold on $\sigma ^m$ ensures that IMFs with a uniformly high $\mu ^m$ are discarded as this does not coincide with transient events.

By comparing the selected IMFs (2–6) to their spectral distribution in Fig. 5b, one can see that the major part of their spectra belongs to the interval $\left[ 100,1000\right]$ Hz. The discarded IMFs on the other hand, have higher (IMF1) and lower (from IMF7 onwards) centers of spectral power. Thus, the frequency range of the selected IMFs coincides with the frequency range of bowel sounds defined in literature, e.g. [13].

#### Peeling information-carrying samples from the selected IMFs

The fractal dimension of the IMFs (IMF-FD) is also used to extract the samples that carry information, i.e. sonic events. Threshold-checking at each sample *k* is used to get the peeled IMFs ($p IMF$):

8 $$\begin{aligned} p IMF^m_k = {\left\{ \begin{array}{ll} IMF^m_k, &{}\text {if } F\!D^m_k-F\!D_{\text {min}} > \sigma ^m\\ 0, &{} \text {otherwise}\, . \end{array}\right. } \end{aligned}$$

**Fig. 8:**
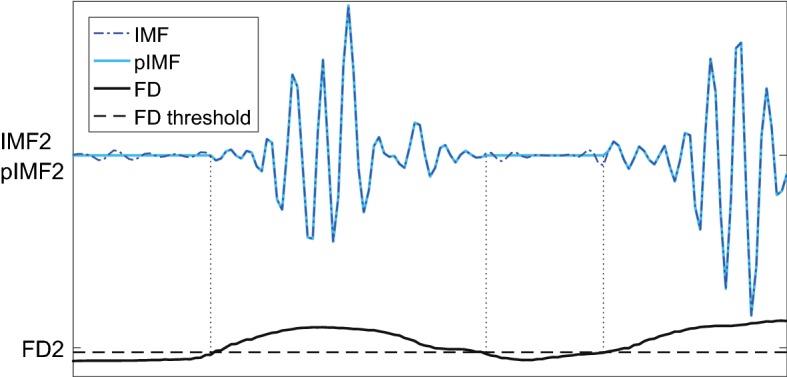
Illustration of peeling information-carrying samples by means of IMF2. Dotted, vertical lines indicate the times where FD2 exceeds its threshold. IMF and pIMF are scaled for better visibility

The *m*-th IMF at sample *k* ($IMF^m_k$) is kept if the local fractal dimension of this IMF, shifted by $F\!D_{\text {min}}$, is larger than the standard deviation $\sigma ^m$ of that IMF. Figure 8 illustrates the peeling: The peeled IMFs have the value of the original IMFs where the IMFs carry information, and are zero otherwise.

This is similar to the iterative fractal dimension-peak peeling algorithm (FD-PPA) by Hadjileontiadis (2005) [18]. However, in the proposed IMF-FD, the information-carrying parts of the IMFs are extracted directly in one step. IMF2–6 are selected in the previous subsection, and their filtered versions feature high correlation with the sonic events in the original signal. IMF1 is discarded even though IMF1 also correlates with the sonic events. This is acceptable when considering the frequency distribution (shown in Fig. 5b) that implies IMF1 to contain high-frequency noise. The IMFs of index 7 and higher have decreasing correlation with the original signal’s information that we are interested in and have been discarded earlier.

#### Combining the peeled samples of selected IMFs

A signal that has been decomposed by an MEMD can be easily recovered by adding the IMFs and the residue together. By selecting specific IMFs and samples, the signal’s content is filtered with respect to both frequencies and time. The filtered sound signal results from the sum of the selected peeled IMFs (that have been processed by Eq. 8):

9 $$\begin{aligned} y^{\text {filtered}}_k = \sum _{m \in \mathbb {M}_S}p IMF^m_k \ . \end{aligned}$$

**Fig. 9:**
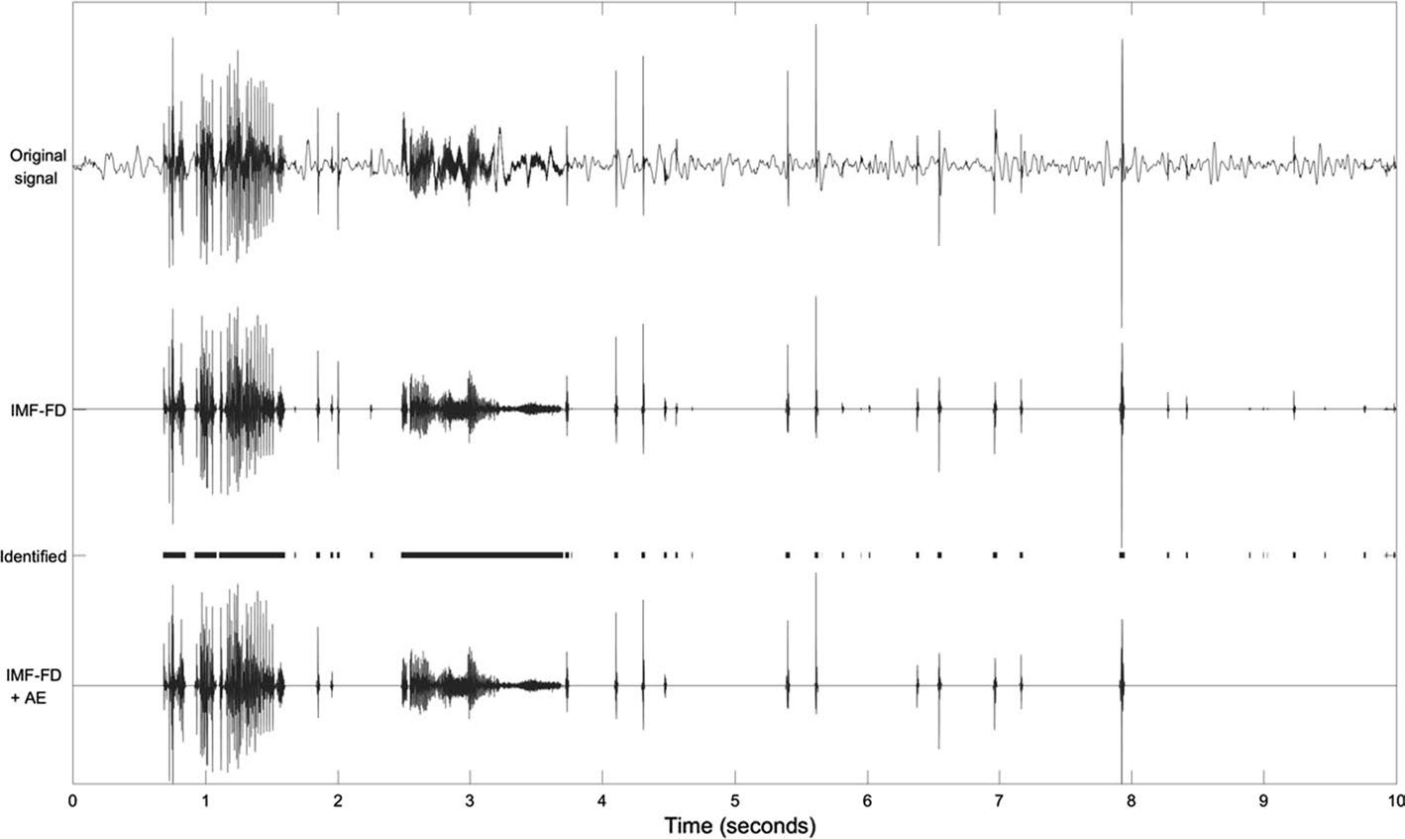
Original and filtered version of the signal in Fig. 1. IMF-FD: intrinsic mode function-based fractal dimension filter. Identified: identified sonic events. IMF-FD + AE: IMF-FD filter plus artifact elimination

For our example, this is shown in Fig. 9. The filtered signal resembles the sonic events of the original signal, and is zero otherwise. Audible analysis of the filtered signal confirms that the IMF-FD method, proposed here, is suitable to enhance the bowel sounds.

#### Proposed method

In summary, the steps of the IMF-FD method for the enhancement of bowel sounds are:

1. Decompose the signal into IMFs using noise-assisted MEMD [24].
2. Generate the sliding-window FDs [23] of the IMFs, Eq. (2).
3. Calculate the mean $\mu ^m$ and the standard deviation $\sigma ^m$ of the FDs, Eqs. (5), (6).
4. Select the IMFs whose $\mu ^m$ and $\sigma ^m$ exceed thresholds, Eq. (7).
5. Peel the sonic events from the selected IMFs, Eq. (8).
6. Sum up the peeled versions of the selected IMFs, Eq. (9).

### Identification of bowel sounds

The sonic events correlate with the intervals where the filtered signal is nonzero. Consecutive sounds separated by less than 10 ms of silence are regarded as a single sonic event. Sounds further apart from each other are counted separately. The identified sonic events are marked in Fig. 9.

#### Artifact elimination

Common artifacts occur in the same frequency range as bowel sounds. It is therefore likely that artifacts are among the identified sonic events. The segment used to illustrate the IMF-FD filter contains bowel sounds in the form of two clusters of bursts and a sparse succession of clicks (Fig. 1). Two exemplary segments of 10 s duration are utilized to describe the artifact elimination: (1) an event of sneezing in an otherwise silent period; (2) a stroke, followed by a cluster of bowel sounds and then noise caused by movements. The stroke is probably caused by hitting the microphone. The bowel sounds occur at lower volume than the artifacts. The chosen segments are only moderately contaminated by stationary background noise.

**Fig. 10:**
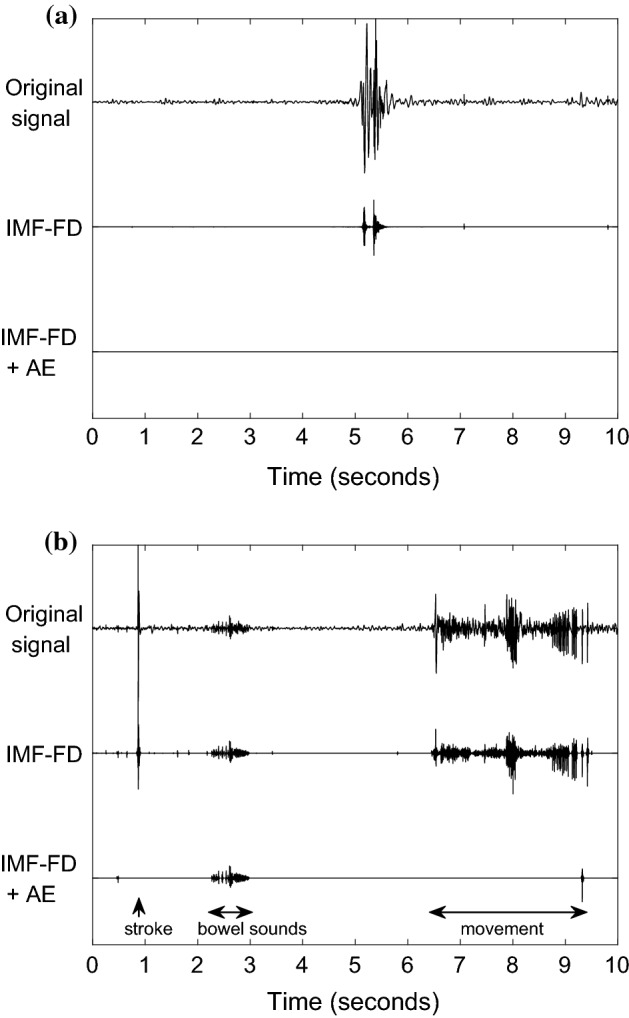
Filtering and artifact elimination. Original and filtered versions of signals contaminated by artifacts. IMF-FD: intrinsic mode function-based fractal dimension filter. IMF-FD + AE: IMF-FD filter plus artifact elimination. **a** Sneezing artifact. **b** Stroke artifact (0.8–1 s), bowel sound (2–3 s), movement artifact (6.5–9.5 s)

Figure 10 presents the original signals and the filtered versions of the artifact-contaminated segments. The artifacts are among the identified sonic events because the most complex IMFs display their non-stationarity.

The absolute power differs between segments, and thus, cannot be used to differentiate artifacts from bowel sounds. The power distribution of the identified sonic events is analyzed in the following to explore the possibility to eliminate these as artifacts. The instantaneous energy density level [19] is non-stationary and can be assigned to instantaneous frequencies.

The total power of a filtered sonic event *j* that is present through the samples $K_j$ is:

10 $$\begin{aligned} P_{\text {tot}}^j = \sum _{k \in K_j}\sum _{m \in \mathbb {M}_S} \left( A^m_k\right) ^2 \ , \end{aligned}$$

i.e., the sum of the squared amplitudes *A* of the Hilbert-Huang spectrum over the selected IMFs and the samples of the identified sound. The power of an identified sound in a certain frequency range $\left[ \underline{f}, \overline{f}\right]$ composes the power of the selected IMFs whose instantaneous frequencies $IF$ lie within the interval at time *k*:

11 $$\begin{aligned} P_{\left[ \underline{f},\overline{f}\right] }^j = \sum _{k \in K_j}\sum _{m \in \mathbb {M}_S} \left( A^m_k(\underline{f}< IF< \overline{f})\right) ^2 \ . \end{aligned}$$

**Fig. 11:**
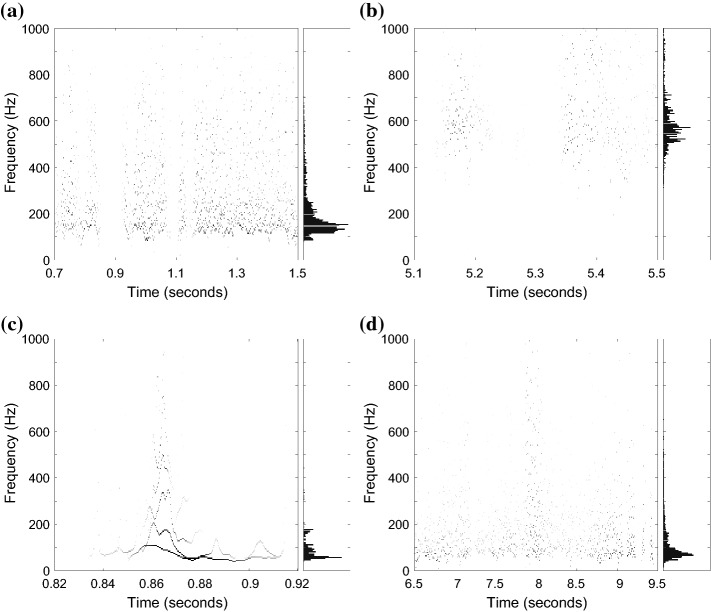
Hilbert-Huang power spectra of selected pIMFs for chosen parts of the examples and power distribution. **a** Cluster of bowel sounds in Fig. 9 (0.7–1.5 s). **b** Sneezing artifact in Fig. 10a (5.1–5.5 s). **c** Stroke artifact in Fig. 10b (0.82–0.92 s). **d** Movement artifact in Fig. 10b (6.5–9.5 s)

Figure 11 presents the Hilbert-Huang power spectra in the frequency range $\left[ 0, 1000\right]$ Hz for chosen parts of the discussed segments. This illustration uses the selected, peeled IMFs as basis. The power of the bowel sounds in Fig. 11a is distributed over the range 80–1000 Hz. Almost no power occurs at frequencies below 80 Hz, and the power is concentrated between 100 and 300 Hz. From the sneezing artifact in Fig. 11b, the IMFs representing frequencies above 400 Hz remain after filtering. The selected IMFs of the artifacts caused by stroke and movement (Fig. 11c, d) contain frequency components in the whole presented range. However, they have more power below 100 Hz compared with the bowel sounds in Fig. 11a.

Based on these observations, the following differentiating power ratios $R^j$ are derived as heuristic rules for artifact elimination:

12 $$\begin{aligned} R^j_{\frac{0-80}{80-1000}} = \, & {} \frac{P_{\left[ 0,80\right] }^j}{P_{\left[ 80,1000\right] }^j} \ , \end{aligned}$$

13 $$\begin{aligned} R^j_{\frac{100-300}{300-1000}} = \, & {} \frac{P_{\left[ 100,300\right] }^j}{P_{\left[ 300,1000\right] }^j} \ . \end{aligned}$$

A sonic event *j* is identified as artifact and eliminated if:

1. The duration is shorter than 20 ms,
2. $$R^j_{\frac{0-80}{80-1000}} > 0.5$$,
3. $$R^j_{\frac{100-300}{300-1000}} < 0.5$$.

Figure 10 summarizes the results of the artifact elimination for the contaminated segments: the main artifacts are kept by the IMF-FD filtering, but they are removed after artifact elimination (IMF-FD + AE). The bowel sounds in Fig. 10b between 2.3 and 5 s are preserved nevertheless. The bottom signal (IMF-FD + AE) in Fig. 9 illustrates that the elimination procedure keeps the bowel sounds also in this example.

### Wavelet-based methods used for comparison

Two filters that enhance bowel sounds based on wavelet transforms are used for comparison: the wavelet transform-based stationary-nonstationary (WTST-NST) filter [15] and the wavelet transform-based fractal dimension (WT-FD) filter [18].

For both WT-filters, the wavelet decomposition is generated with the Matlab function wavedec using Daubechies wavelets of order 8.

The WTST-NST filter is used with the accuracy levels reported by [15]: parameters $F_{\text {adj}} = 3$, $\epsilon = 0.00001$. To ensure freedom of boundary effects, however, the resolution scale is restricted to one level less than the maximum level of wavelet decomposition calculated by means of the Matlab function wmaxlev.

The WT-FD filter is implemented with the fractal dimension according to Sevcik [23], a sliding window size of $W_L = 0.0006 \cdot F_s+1$, and the parameters $\epsilon = 0.01$ and $\text {acc} = 0.01$ for the stopping criterion and the accuracy level of the peak peeling algorithm, respectively. These tuning parameters are based on the original publication [25]. In order to analyze the fractal dimension of one-sample shifted vectors of length *N* in the WT-FD filter, the length of the sliding windows should be $W_L<< N$ [18]. The lowest ratio $N/W_L$ in [25] is 34 (for the shortest signals with 2048 samples, fixed sliding window length $W_L = 30$, and fixed resolution scale $M=1$). Based on that, the maximum resolution scale is $M = 5$ corresponding to a minimum ratio $N/W_L$ of around 50 (for the signals with 40,000 samples, a fixed sliding window length $W_L = 25$, and a fixed resolution scale $M=5$).

## Results

### Qualitative comparison of IMF-FD filter and wavelet-based methods

**Fig. 12:**
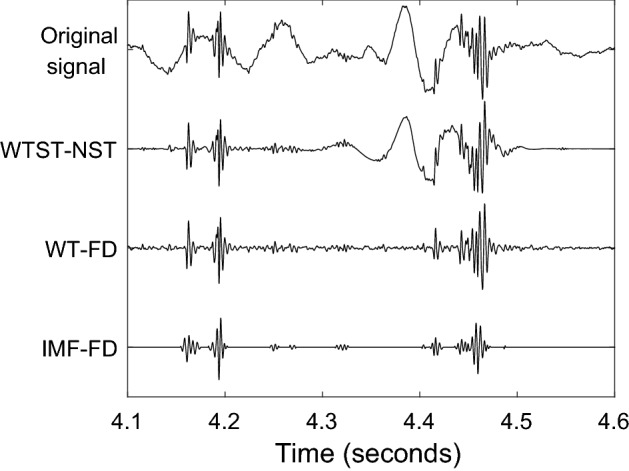
Original and variously filtered signals. WTST-NST: wavelet transform-based stationary-nonstationary filter [15]. WT-FD: wavelet transform-based fractal dimension filter [18]. IMF-FD: intrinsic mode function-based fractal dimension filter

The training examples were only moderately contaminated by background noise. A part of a more severely contaminated segment is presented in Fig. 12. Figure 12 compares the IMF-FD, WTST-NST and WT-FD filters. All filters successfully extract the instances of the signal with higher local frequencies. The WTST-NST preserves modes with lower frequency that are removed by the other two filters. An important advantage of the proposed IMF-FD method is that it results in a zero signal except for the locations of sonic events. This enables a more robust and automated identification of sonic events. Neither WTST-NST nor WT-FD allow such an easy identification of relevant events but rather require further thresholds at this stage.

**Fig. 13:**
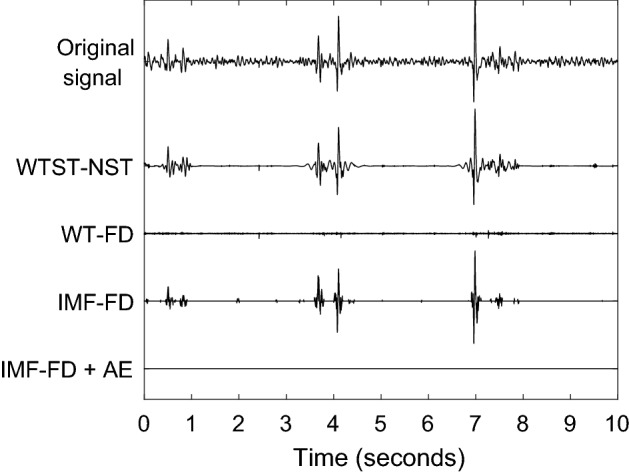
Example of a segment without bowel sounds but other non-stationary artifacts. Original and variously filtered signals. WTST-NST: wavelet transform-based stationary-nonstationary filter [15]. WT-FD: wavelet transform-based fractal dimension filter [18]. IMF-FD: intrinsic mode function-based fractal dimension filter; IMF-FD + AE: IMF-FD filter plus artifact elimination

Figure 13 reinforces the strength of the IMF-FD filter in enhancing the most prominent non-stationary sonic events with frequency variations. This figure presents a segment without bowel sounds but with other non-stationary events. The events are dampened significantly by the WT-FD method, but still present after filtering by WTST-NST and IMF-FD. However, all falsely identified events are removed after processing the IMF-FD filtered events by the proposed artifact elimination.

### Identification of bowel sounds and artifact elimination in test set

The IMF-FD filter with subsequent artifact elimination was applied to the test set. Detected sonic events were compared to the labeled bowel sounds.

**Fig. 14:**
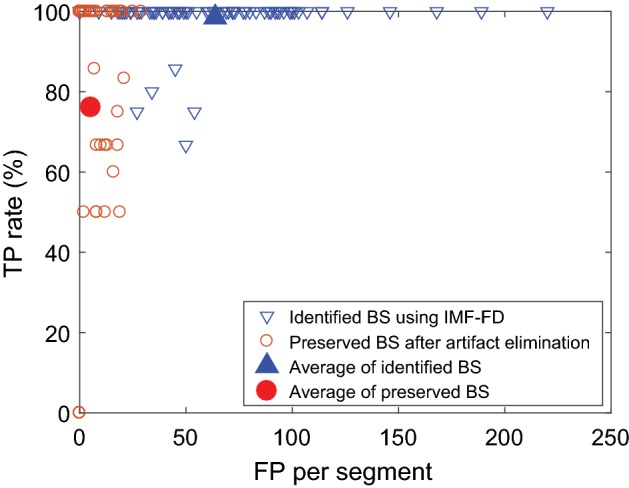
True positive (TP) rate vs. false positive (FP) detections per segment for the 80 segments in the test set

Sonic events in the test set with 80 segments were identified using the IMF-FD filter and compared to the labeled bowel sounds. Figure 14 summarizes the results: Each marker indicates the rate of true positive (TP) detections to the number of false positive (FP) detections for one segment. If one labeled BS is identified as several BS, it is counted as a single TP. If a segment contains no labeled bowel sounds, the TP rate (number of TP BS divided by number of labeled BS) was set to 100%. This applies to 44 segments. Taking this into account, all labeled bowel sounds are identified in 75 of the 80 analyzed segments. In the remaining five segments, 67% to 86% of the labeled bowel sounds are identified. The sensitivity to bowel sounds is high, but there are also artifacts identified: The number of FPs lies between 0 and 220 per segment, mean (std) = 63.8 (40.4). High numbers of FPs occur for “silent” segments without predominant non-stationary sonic events. Since no event dominates the IMFs, the most “complex” parts of silent signals are equally distributed and lead to this many FPs.

The proposed artifact elimination reduces the number of false detections significantly: 0–29 FPs remain per segment, mean (std) $=$ 5.3 (7.5). In the segment for which 220 FPs were detected originally (the highest number), all FPs are eliminated. However, the TP rate decreases at the same time: A 100%-detection rate is retained for 52 of 75 segments. The mean TP rate of all segments decreases from 98.5% to 76.1% after artifact elimination.

## Discussion

The proposed IMF-FD method enhances and simultaneously isolates non-stationary transient sounds from stationary background noise. The simultaneous isolation is an advantage compared to the WTST-NST and WT-FD filters. Moreover, the data driven decomposition of the signal by MEMD makes the filter more flexible, while the wavelet based filters assume predefined basis functions.

The manual marking of bowel sounds in the test segments is challenging. First, one tends to “hear” more subtle events the more silent the overall signal is. Second, the exact beginning and end of a given sonic event is ambiguous. Third, when bowel sounds occur in clusters or in close succession, one has to decide if they are counted as one or several events. In some test segments, the bowel sounds were more clear and distinguishable than in others. This adds uncertainty to the manual labeling of bowel sounds. Nevertheless, in 94% of the tested 10 s-segments, all labeled bowel sounds were detected by the IMF-FD.

A higher degree of contamination by artifacts challenges the identification of real bowel sounds. Many typical noise sources in abdominal recordings cause sounds in the same frequency range as BS. The duration and power distribution of sonic events has been used before to differentiate bowel sounds from artifacts [13]. The developed set of heuristic rules worked well for the tuning set, and eliminated typical artifacts such as clothes swishing over the microphone. From the test set, some initially identified BS were eliminated as well. Some of these sonic events may have been falsely labeled as bowel sounds during the manual, audio-visual analysis. It may also be helpful to refine the artifact elimination. However, the examples showed that the IMF-FD filtering retains the original frequency composition of segmented events which can be utilized to eliminate artifacts, for example by simple heuristic rules.

By selecting the IMFs based on their relative complexity, the most prominent non-stationary events in the analyzed signal are extracted. No tuning to different frequency ranges is needed which adds flexibility as compared to the existing methods and facilitates the use of the IMF-FD filter in other applications. On the other hand, the IMF-FD filter will enhance the most prominent non-stationary events in the processed signal. If the signal has no such events, false events may be segmented. This limitation caused more than 200 FPs in one of the tested segments. To reduce this effect, additional criteria could be used to select the relevant IMFs, such as the instantaneous frequencies if the target frequency range is known a priori. Another measure to mitigate this behavior could be to superimpose a signal with a known event that does not interfere with the components of the analyzed signal.

One might argue that the computational time needed for the MEMD may be too long for real-time applications. However, bowel sound analysis with the purpose of diagnosing diseases is usually not time-critical. Assuming that patients carry a recording device for a certain time, gastrointestinal diseases can be diagnosed off-line. Furthermore, the MEMD can be terminated after sifting out a defined number of IMFs that have been proven to be relevant. The criteria for selecting IMFs must be refined in this case.

Bowel sounds are not necessarily present in every segment, nor is it given that the noise remains within limits that allow a BS detection. No pre-processing is required for moderately contaminated recordings. Nevertheless, prominent peaks in the frequency spectrum affect the analysis of the power distribution over the frequency range and challenge the artifact elimination. It might be a good idea to use notch filters to remove noise caused by electric interferences.

Artifacts will be more prominent in real life situations compared to abdominal sound monitoring in the clinic. The tested artifact elimination based on heuristic rules reduced the TP rate in some occasions of the test set by up to 50%. More advanced strategies to separate the interesting events from background noise and artifacts will be needed. Pattern recognition methods might be applicable to subsequently analyze the extracted information on bowel sounds. The eventual diagnosis of gastrointestinal diseases or identification of digestive periods are out of the scope of this paper and left for future work.

## Conclusion

The proposed method successfully enhances and segments transient bowel sounds from background noise. The dyadic filter bank property ensures that characteristic frequency components are identified. The method was tested on contaminated recordings. With a detection rate of almost 100%, the enhancement of bowel sounds was successful. The high sensitivity towards non-stationary events is connected with a lower selectivity to bowel sounds, and some artifacts were identified as well. The artifacts were subsequently eliminated based on the power distribution over particularly interesting frequency ranges. A statistical artifact elimination trained on a larger data set with various bowel sounds and types of artifacts may be used in future work to enhance the elimination performance.
